# Thioredoxin1 Binding Metastasis-Associated Lung Adenocarcinoma Transcript 1 Attenuates Inflammation and Apoptosis after Intracerebral Hemorrhage

**DOI:** 10.14336/AD.2023.0507

**Published:** 2024-05-07

**Authors:** Ru Chen, Qi Xie, Lexing Xie, Jiacheng Huang, Linlin Hu, Hui Lu, Peixia Shi, Qian He, Qin Zhang, Changxiong Gong, Shuang Zhang, Bingqiao Wang, Guoqiang Yang, Qingwu Yang

**Affiliations:** Department of Neurology, Second Affiliated Hospital, Army Medical University (Third Military Medical University), Chongqing 400037, China

**Keywords:** Thioredoxin1, RNA binding protein, intracerebral hemorrhage, inflammation

## Abstract

Post-transcriptional regulation and RNA-binding proteins (RBPs) play vital roles in the occurrence of secondary injury after intracerebral hemorrhage (ICH). Therefore, we identified RBPs distinctively expressed after ICH by screening and determined thioredoxin1 (Txn1) as one of the most distinctive RBPs. We employed an ICH model and in vitro experiments to investigate the role of Txn1 in ICH. Firstly, we found that Txn1 was mainly expressed in microglia and neurons in the central nervous system, and its expression was significantly reduced in perihematomal tissue. Additionally, adeno-associated virus (AAV) carrying Txn1 was injected into the ICH rat model. Our results showed that overexpression of Txn1 reduced secondary injury and improved outcome in the ICH rat model. Moreover, to understand the therapeutic mechanism of Txn1 after ICH, we performed RNA immunoprecipitation combined with high-throughput sequencing. The results showed that Txn1 binds to inflammation- and apoptosis-related mRNAs and affects gene expression through RNA splicing and translation. Finally, RNA pull-down assays and in vitro experiments confirmed that Txn1 binds to metastasis-associated lung adenocarcinoma transcript 1 (MALAT1), leading to reduced inflammation and apoptosis. Our study suggests that Txn1 is a potential therapeutic target for alleviating ICH-induced brain injury.

## INTRODUCTION

Intracerebral hemorrhage (ICH) refers to the non-traumatic rupture of blood vessels in the brain, which results in blood accumulation in the brain parenchyma [1]. Previous studies have proven that inflammation and apoptosis critically affect the outcome of ICH [2]. Although the pathological process of ICH has been investigated, therapeutic interventions still have limitations. An explanation for this is that previous studies mainly focused on the pathological processes of secondary injury after ICH and ignored the gene regulation of these pathological processes. Post-transcriptional regulation refers to the modification and regulation of the gene at the post-transcriptional level, which mainly regulates RNA splicing, polyadenylation, mRNA stability, mRNA localization, and translation [3], and it is inseparable from RNA-binding proteins (RBPs).

Previous studies have shown that post-transcriptional regulation of genes and RBPs are closely associated with ICH. Dykstra-Aiello et al. demonstrated that differential alternative splicing was associated with vascular and brain injury after ICH [4]. Furthermore, Shen et al. found that RBPs bind to Ca^2+^/calmodulin-dependent protein kinase II RNA, leading to intracellular-Ca^2+^ overload and neuronal degeneration, aggravating brain injury [5]. Feng et al. also revealed the close relationship between RBP and secondary injury of ICH [6]. Therefore, it is practicable to control the pathological process of ICH by intervening in post-transcriptional regulation through RBPs.

In our study we adopted an ICH model combined with RNA sequencing (RNA-seq) technology to identify differentially expressed RBPs, and thioredoxin1 (Txn1) was one of the most differentially expressed RBPs. Txn1 is a member of thioredoxins (Trxs) family and has been characterized as a guard of the intracellular redox state and antioxidants [7]. Previous studies have shown that Txn1 is involved in liver cancer, myocardial ischemia, pulmonary fibrosis, and brain diseases such as intracerebral infarction and Alzheimer's disease [8-12]. It mainly relieves oxidative stress in the central nervous system through Nrf2/Txn1/TXNIP [11] and other signal pathways, thus reducing brain injury. However, its specific function in ICH remains unclear.

In this study, we employed an ICH rat model coupled with in vitro experiments, to investigate the effect and mechanism of Txn1 in brain injury after ICH. Our findings showed that overexpression of Txn1 significantly inhibited inflammation and apoptosis and alleviated functional impairment after ICH. Furthermore, using RNA immunoprecipitation coupled with high-throughput sequencing (RIP-seq) technology and RNA pull-down assays, we confirmed that the protective effect of Txn1 in brain injury could be associated with the binding of the long noncoding RNA (lncRNA) metastasis-associated lung adenocarcinoma transcript 1 (MALAT1), thereby attenuating inflammation and apoptosis after ICH.

## MATERIALS AND METHODS

### Animals

Sprague-Dawley (SD) rats (male, 13 months of age) were purchased from the SJA Laboratory Animal Company (Hunan, China) and housed in the Animal Center of Xinqiao Hospital, the Third Medical Military University, Chongqing, China, with free access to food and water. All experiments were conducted following the protocols of the Laboratory Animal Welfare and Ethics Committee of the Army Medical University (AMUWEC20226329) (Chongqing, China).

### Generation of the ICH Model

The ICH model was developed using a previous method [13]. Briefly, a syringe pump (KD Scientific, Holliston, MA) was used to inject 70 µL of autologous blood or saline into the left striatum of rat (3.2 mm lateral and 0.8 mm anterior of the bregma at a depth of 5.4 mm) at a rate of 10 µL/min. The needle was left in the brain for 7 min after injection. Finally, the needle was slowly removed, and the head’s skin was carefully sutured.

### Injection of Adeno-associated virus (AAV) carrying Txn1 in the rat

5 µL AAV (HBAAV2/9-CMV-r-Txn1-3xflag-zsGreen) carrying Txn1 or vector (HBAAV2/9-CMV-zsGreen) (Hanbio, Shanghai, China) was injected at a flow rate of 1 µL/min into the left striatum of rat (3.2 mm lateral and 0.8 mm anterior of the bregma at a depth of 5.4 mm).

### Immunofluorescence Staining

Brain tissue was cut into 30-µm sections and incubated in phosphate-buffered saline (PBS), which included 0.5% Triton X-100 and 10% donkey serum for 1 h at 37°C. Subsequently, the following primary antibodies were incubated with brain sections for 12 h at 4°C: anti-GFAP (1:100, Cat#: ab53554, Abcam, Cambridge, UK), anti-NeuN (1:200, Cat#: ab104224, Abcam, Cambridge, UK), anti-Iba1 (1:100, Cat#: ab5076, Abcam, Cambridge, UK), and anti-Txn1 (1:100, Cat#: PA5-95589, Invitrogen, Carlsbad, CA). The brain sections were then incubated with the fluorescent secondary antibody, including Alexa Fluor 555 donkey anti-goat (1:400, Cat#: A32816, Invitrogen, Carlsbad, CA), Alexa Fluor 555 donkey anti-mouse (1:400, Cat#: A32773, Invitrogen, Carlsbad, CA), Alexa Fluor 555 donkey anti-rabbit (1:400, Cat#: A32794, Invitrogen, Carlsbad, CA), Alexa Fluor 488 donkey anti-rabbit, (1:400, Invitrogen, Cat#: A32790), Alexa Fluor 488 donkey anti-mouse (1:400, Cat#: A21202, Invitrogen, Carlsbad, CA), Alexa Fluor 488 donkey anti-goat (1:400, Cat#: A32814, Invitrogen, Carlsbad, CA), and were washed with PBS for 10 min. The cell nuclei were stained for 20 min with 4’,6-diamidino-2-phenylindole (DAPI) (1:3000, Sigma Aldrich, St Louis, MO). Finally, the sections were washed with PBS for 30 min and mounted. The section without the primary antibody was used as the negative control and the liver section as the positive control. A confocal fluorescence microscope (Leica Sp5; Leica Microsystems, Mannheim, Germany) was used to image the sections. The mean of positive cells under three microscopic vision fields (200x) was determined using ImageJ software.

### Quantitative Real-Time PCR

Total RNA was extracted from brain tissues or cells using TRIzol reagent (Sigma Aldrich, St Louis, MO) and reverse-transcribed to cDNA (TaKaRa, Shiga, Japan). Next, qRT-PCR was performed using a Thermo Fisher PCR instrument (Thermo Fisher Scientific, Waltham, MA). Shanghai Sangon Biotechnology Co., Ltd. was employed to synthesize the primers (Table 1).

**Table 1:** Primers used for quantitative real-time polymerase chain reaction (qRT-PCR).

Primers	Forward	Reverse
**Rat *IL-10***	5’-AAAGCAAGGCAGTGGAGCAG-3’	5’-AGTAGATGCCGGGTGGTTCA-3’
**Rat *IL*-6**	5’-AGGAGTGGCTAAGGACCAAGACC-3’	5’-TGCCGAGTAGACCTCATAGTGACC-3’
**Rat *TNF-α***	5’-GCATGATCCGAGATGTGGAACTGG-3’	5’-CGCCACGAGCAGGAATGAGAAG-3’
**Rat *GAPDH***	5’-TCCCTCAAGATTGTCAGCAA-3’	5’-AGATCCACAACGGATACATT-3’
**Rat Txn1**	5’-ATCAAGCCCTTCTTTCATTCCCTCTG-3’	5’-CAGCAACATCCTGGCAGTCATCC-3’
**Rat MALAT1**	5’-TGCAGTGTGCCAATGTTTCG-3’	5’-GGCCAGCTGCAAACATTCAA-3’
**Mouse *IL-6***	5’-CTCCCAACAGACCTGTCTATAC-3’	5’-CCATTGCACAACTCTTTTCTCA-3’
**Mouse *TNF-α***	5’-ATGTCTCAGCCTCTTCTCATTC-3’	5’-GCTTGTCACTCGAATTTTGAGA-3’
**Mouse *GAPDH***	5’-GGTTGTCTCCTGCGACTTCA-3’	5’-TGGTCCAGGGTTTCTTACTCC--3’
**Mouse Txn1**	5’-TTCCCTCTGTGACAAGTATTCC -3’	5’-TCAAGCTTTTCCTTGTTAGCAC -3’
**Mouse MALAT1**	5’-TGTGTGGCAAGAATCAAGCAAG-3’	5’-TCCAACAAGGTGTTACGGTAGG -3’
**MALAT1 siRNA**	5’-GAAAGAAGCAAGAUAGAAATT-3’	5’-UUUCUAUCUUGCUUCUUUCTT -3’

MALAT1: metastasis-associated lung adenocarcinoma transcript 1; Txn1: Thioredoxin1; GAPDH: Glyceraldehyde 3-phosphate dehydrogenase

### Western Blot

Perihematomal tissue and cell proteins were extracted using protein lysate (Beyotime, Shanghai, China) and separated by 12% sodium dodecyl sulfate (SDS) polyacrylamide gel electrophoresis (SDS-PAGE) (Beyotime, Shanghai, China). Next, the proteins were placed onto polyvinylidene fluoride membranes (Millipore, Burlington, MA) by electroblotting. Subsequently, the membranes were incubated with primary antibodies for 12 h at 4°C; the antibodies included Txn1 (1:500, Cat#: PA5-95589, Invitrogen, Carlsbad, CA), β-actin (1:500, Cat#: SC47778, Santa Cruz, Dallas, Texas), B-cell lymphoma protein 2 (Bcl-2) (1:500, Cat#: ab196495, Abcam, Cambridge, UK), and Bcl-2 associated X protein (Bax) (1:500, Cat#: SC7480, Santa Cruz, Dallas, Texas). Membranes were washed and incubated with horseradish peroxidase (HRP)-conjugated secondary antibodies (goat anti-rabbit or anti-mouse) at room temperature for 1.5 h. Finally, the enhanced chemiluminescence (ECL) substrate and the ImageJ software (version 1.4.3.67) were used to visualize the membranes and quantify the gray values, respectively.

### Hematoxylin and Eosin (H&E) Staining

Brain tissues were dissected and fixed in paraformaldehyde using an established methodology [14]. Then, the tissues were embedded in paraffin and cut into 5-µm sections and mounted on slides. Next, the slides were washed with 90% and 70% ethanol solutions. Finally, the slides were stained and observed. Perihematomal tissue at the striatal section was shown as the representative position.

### Nissl Staining

Nissl staining was performed following a previous method [15]. Briefly, brain tissue was cut and mounted, and the slides were stained with thionin (Sigma-Aldrich, St Louis, MO). Finally, the slides were observed after alcohol dehydration. Therefore, the number of perihematomal-positive cells at the striatal section in three standardized microscope fields of two consecutive sections was counted and analyzed to quantify the staining results.

### Fluoro-Jade B (FJB) Staining

Following an established methodology [16], 5-μm thick brain tissue sections were immersed in graded alcohols and washed in distilled water. Next, the sections were sealed with 0.06% KMnO4, hatched in a 0.01% FJB solution (Millipore, Burlington, MA) for 40 min, and washed. Subsequently, the sections were dehydrated and washed. Finally, perihematomal-positive cells at the striatal section of three standardized microscope fields of two consecutive sections were counted and analyzed.

### Terminal Deoxynucleotidyl Transferase dUTP Nick End Labeling (TUNEL) staining

Following an established methodology [17], neuronal apoptosis was detected using a colorimetric TUNEL Apoptosis Assay Kit (Beyotime, Shanghai, China) according to the manufacturer’s instructions. Next, three visual fields of the perihematomal brain tissue at the striatal section from two consecutive sections were randomly selected for detection, and the mean values of positive cells were calculated for statistical analysis.

### Hematoma Measurements

The brains were dissected and fixed after perfusion according to a previously reported procedure [18]. Next, the tissues were embedded in an optimal cutting temperature (OCT) compound (Sakura, Tokyo, Japan) and sectioned using a freezing microtome. The tissue was then cut into consecutive sections (2 mm thick) and sequentially mounted on slides. Finally, ImageJ software was used to determine the ICH volume in each section.

### Brain Water Content (BWC)

The brain tissue was dissected and then heated to 60°C for 24 h. The tissue was weighed and recorded before and after the heat treatment. Finally, we calculated the BWC using the following formula: ([wet tissue weight - dry tissue weight]/wet tissue weight) × 100%.

### Survival Analysis

The number of dead rats were counted after ICH induction. The survival rate of each group was determined as follows: (number per group - number of dead rats per group)/number per group. Subsequently, the Kaplan-Meier survival analysis was performed using GraphPad Prism 8.

### Cell Culture and Transfection

BV2 cell line was purchased from Procell Life Science & Technology Co., Ltd. (Wuhan, China). The cells were maintained in an incubator containing 5% CO2 and 95% atmospheric air at 37°C. Subsequently, the medium was replaced every 24 h, and the cells were sub-cultured or cryopreserved when the cell density reached 70-80%.

MALAT1 siRNA combined with Lipofectamine 3000 reagent (Invitrogen, Carlsbad, CA), lentivirus (LV) carrying Txn1 (HBLV-m-Txn1-3xflag-ZsGreen-PURO), or the empty vector (HBLV-ZsGreen-PURO) were used to transfect BV2 cells following the manufacturer's instructions. The cells were assigned to the following four groups: empty vector, LV-Txn1, MALAT1 siRNA + empty vector, and MALAT1 siRNA + LV-Txn1. The transfection efficiency was subsequently detected by qRT-PCR and western blot (WB).

### ICH In vitro Model

BV2 cells were cultured in 6-well plates with Dulbecco’s Modified Eagle Medium (DMEM) as described in the previous method [19]. Hemoglobin (Sigma Aldrich, St Louis, MO) stimulation was applied to the plates (20 μM per well) to illustrate the in vitro ICH model at 37°C and 5% CO2 for 6 h. Finally, the cells were washed with PBS and collected.

### RNA Immunoprecipitation and High-throughput Sequencing (RIP-seq)

Hela cells were collected and lysed in a lysis buffer for DNA digestion. The lysate was then incubated with the antibody overnight at 4°C for immunoprecipitation. Subsequently, the magnetic beads were resuspended and eluted. Next, the samples were subjected to the enzymatic action of the FastAP enzyme, T4 polynucleotide kinase (T4 PNK) enzyme, and proteinase K. The cDNA libraries were established by the Illumina ScriptSeq™ v2 RNA-Seq Library Preparation Kit (Epicentre, San Diego, CA). The cDNAs were purified and amplified and PCR products corresponding to 200-500 bps were purified, quantified, and stored at -80 °C until used for sequencing.

### RNA Pull-down Assays

RNA was synthesized based on a previously established method using a MAXIscript Kit (Thermo Fisher Scientific, Waltham, MA) following the manufacturer’s instructions [20]. Next, a Pierce Magnetic RNA-Protein Pull-Down Kit (Thermo Fisher Scientific, Waltham, MA) was used to evaluate the relationship between the RNA and proteins. After that, the RBP complexes were washed and eluted. Finally, the samples were analyzed using mass spectrometry (MS) (5600-plus, AB SCIEX, Toronto, Canada) and western blot.

### Modified Neurological Severity Score

We calculated the modified neurological severity score (mNSS) of sham rats, AAV-Txn1 injected ICH rats, empty vector injected ICH rats and ICH rats on days 1, 3, 7, 14, and 21 after ICH, following the guidelines in Table 2. The higher scores represent more severe neurological defects. We also used corner tests for identifying sensory and postural asymmetry before ICH as a baseline and on ICH 1, 7, 14, and 21 days. Briefly, we used two plates to form a 30 corner and the direction the rat faced when turning the corner was recorded. The result was shown as the percentage of left turns.

### Flow Cytometry

Annexin V-FITC cell apoptosis detection kit (Cat#C1062M, Beyotime, Shanghai, China) and DAPI (Cat#C1002, Beyotime, Shanghai, China) were purchased and the staining process was performed following the instructions. Gallios flow cytometry (Beckman Coulter, Pasadena, California) was used to divide cells into four groups and the proportion of corresponding cell groups was detected.

**Table 2:** Modified Neurological Severity Scores (mNSS).

Test items	Score
**Raising the rat by the tail (normal = 0, maximum = 3)**	(3)
**Flexion of forelimb** **Flexion of hindlimb** **The head moved > 10^o^ to the vertical axis within 30 s**	1 1 1
**Placing rats on the floor (normal = 0, maximum = 3)**	(3)
**Normal walk** **Inability to walk straight** **Circling toward the paretic side** **Falls to the paretic side**	0 1 2 3
**Sensory tests (normal = 0, maximum = 6)**	(6)
**Balances with a steady posture** **Grasps side of the beam** **Hugs the beam and one limb falls from the beam** **Hugs beam and two limbs fall from the beam or spin on the beam (> 60 s)** **Attempts to balance on beam but falls off (> 40 s)** **Attempts to balance on beam but falls off (> 20 s)** **Falls off, no attempt to balance or hang on to beam (< 20 s)**	0 1 2 3 4 5 6
**Reflex absence and abnormal movements**	(4)
**Pinna reflex (head shakes when auditory meatus is touched)** **Corneal reflex (eye blink when the cornea is lightly touched with cotton)** **Startle reflex (motor response to a brief noise)** **Seizures, myoclonus, myodystony**	1 1 1 1

### Statistical Analysis

Statistical analysis was performed using Graph Pad Prism 8.0. The Shapiro-Wilk test was performed to assess the normality of the distribution of data. Normally distributed data are presented as mean±standard error and non-normal distribution data are presented as the median and interquartile ranges (IQR). For normally distributed data, unpaired, 2-tailed Student’s t test was performed for comparisons between two groups, and one-way ANOVA was performed for multiple comparisons. For non-normally distributed data, the Mann-Whitney U test was performed for the comparisons between the two groups and the Kruskal-Wallis test was performed for multiple comparisons. The values of *P < 0.05, **P < 0.01 were considered statistically significant.

## RESULTS

### Txn1 was the Most Differentially Expressed RBP Associated with Secondary Injury after ICH

We used two age and sex-matched rats to establish ICH model. RNA sequencing was performed using perihematomal and contralateral brain tissue at the same location as the control in both groups. Based on the assessment of the principal component analysis (PCA), the profile of the ICH group was shifted compared to the sham group. The results suggested that ICH induced significant differential expressions of genes compared with the sham group (Supplementary Fig. 1A). Additionally, 1965 up-regulated genes and 177 down-regulated genes were identified after ICH (Supplementary Fig. 1B). These up-regulated genes were dominantly linked with secondary injury after ICH, such as positive regulation of cytokine production and regulation of lymphocyte activation (Supplementary Fig. 1C). The top ten expressed RBPs were mapped and suggested that most RBPs were upregulated after ICH (Supplementary Fig. 1D). Furthermore, we screened 142 differentially expressed genes, which were related to inflammation and immune response reported by previous studies [21-24] and found that two inflammatory response-related RBP genes were differentially expressed after ICH (Supplementary Fig.1E): Txn1 and tripartite motif containing 25 (Trim25). Compared with Trim25, the differential expression of Txn1 was more obvious after ICH. Therefore, we selected Txn1 for further study (Supplementary Fig. 1D, F).

### Txn1 was Decreased after ICH

**Figure 1.**
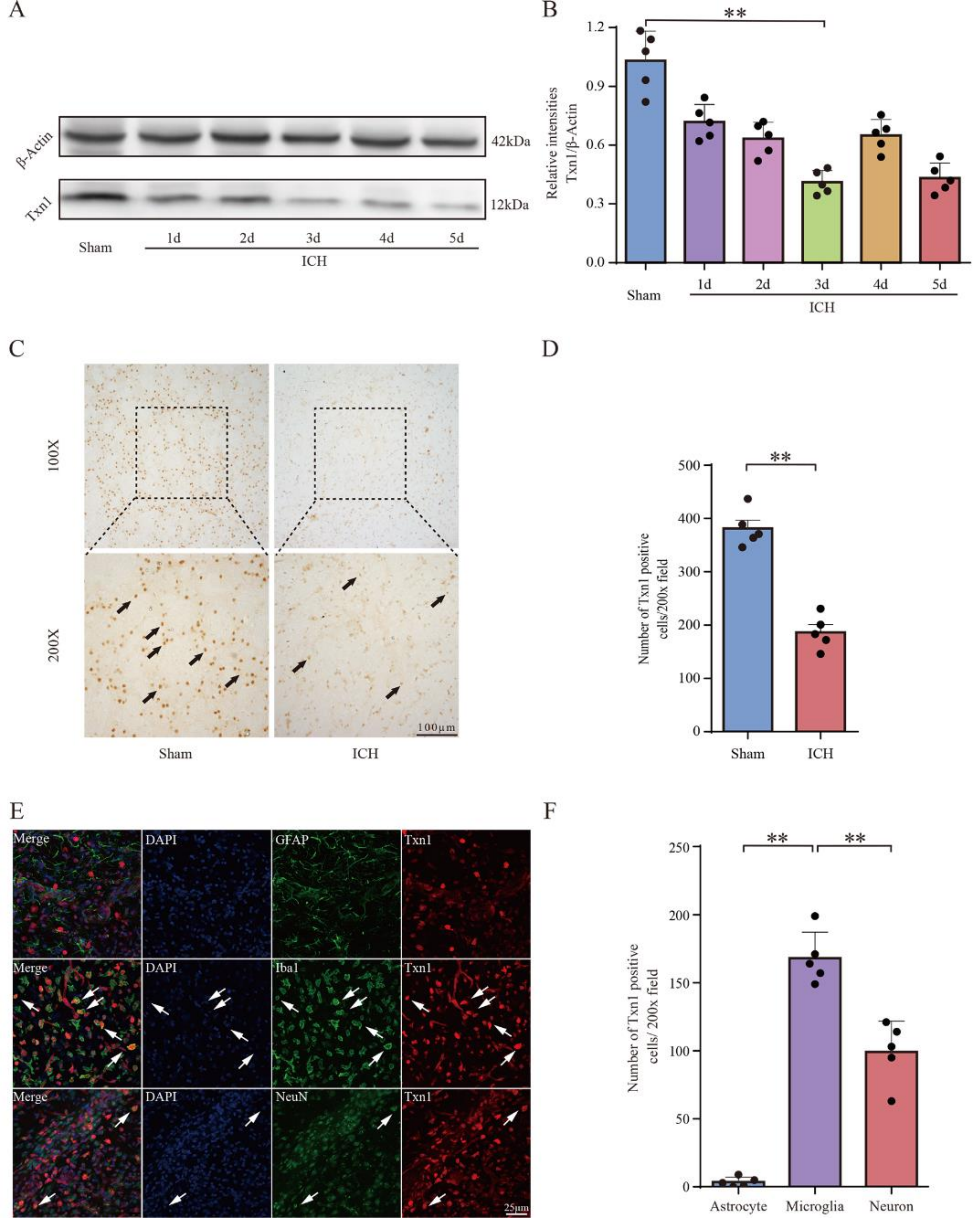
**Txn1 was significantly decreased in rats after ICH**. (**A**) Expression of Txn1 in the perihematomal area detected by Western blotting analysis. (**B**) The relative intensities of Txn1 expression level (Txn1/b-actin) in the perihematomal area (n=5). (**C**) Txn1 expression in perihematomal tissue detected by immunohistochemistry 3 days after ICH. The arrows indicated Txn1 positive cells, Scale bar =100μm. (**D**) The number of Txn1 positive cells in the perihematomal area (n=5). (**E**) Expression of Txn1 in perihematomal striatum detected by immunofluorescence at 3 days after ICH. The arrows indicated Txn1 positive cells. Scale bar = 25μm. (**F**) Immunostaining results showed Txn1^+^/NeuN^+^, Txn1^+^/Iba-1^+^, and Txn1^+^/GFAP^+^ cells in the perihematomal area (200x field, n=5). Data in (B), (D), and (F) were shown as mean ± SEM. One way ANOVA was performed for data comparisons in (B) and (F). Unpaired t-test was performed for comparison of means between two groups in (D). Statistical significance was denoted by **P < 0.01.

We employed western blots to analyze the protein levels after ICH. Interestingly, compared with the sham group, the protein level of Txn1 was significantly decreased (Fig. 1A, B), which was dramatically different from the mRNA expression of Txn1 (Supplementary Fig. 2A). We speculated that this may be due to Txn1 depletion and ubiquitination after ICH [25, 26]. Since Txn1 fulfills its function as a protein, we selected three days after ICH as the time point for subsequent experiments, following the lowest Txn1 protein level. Next, we performed immunohistochemical staining and found the number of Txn1-positive cells was reduced in the striatum after ICH induction (Fig. 1C, D). The immunofluorescence staining showed that Txn1 was colocalized with NeuN^+^ neurons and Iba1^+^ microglia (Fig. 1E, F) in the perihemetomal tissue. The results implied that Txn1 may contribute to neuronal damage and inflammatory immune response after ICH.

**Figure 2.**
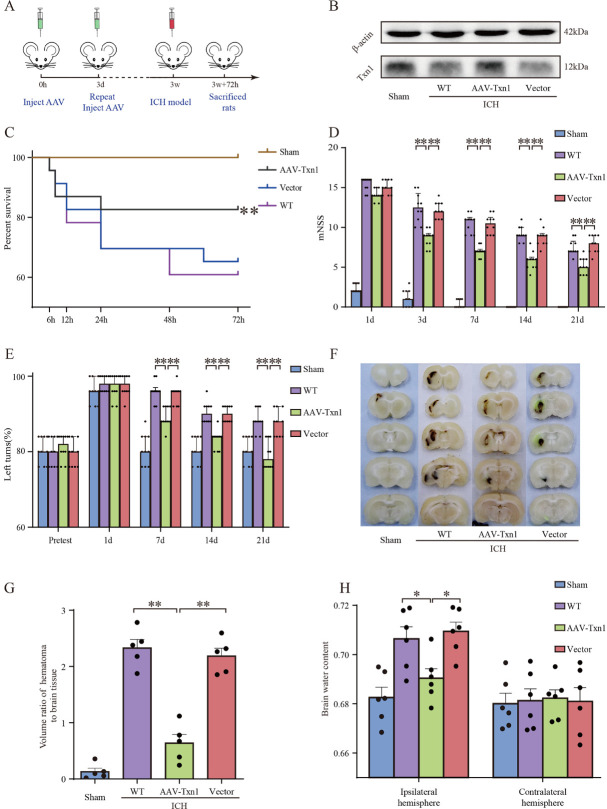
**Txn1 attenuated brain edema, and improved neurological function of rats after ICH**. (**A**) Experimental workflow of injection AAV-Txn1 in rats and establish ICH rat model. (**B**) Western blotting analysis showed expression of Txn1 in rats. (**C**) Survival statistics of the sham rats, AAV-Txn1 rats after ICH, empty vector rats after ICH and WT rats after ICH (n=10). (**D**) Modified neurological severity score (mNSS) of the four groups on days 1, 3, 7, 14, and 21 (n=10). (**E**) The percentage of left turns of four groups before ICH and 1, 7, 14, and 21 days after ICH (n=10). (**F**) The brain tissue sections of the four groups. (**G**) The volume ratio of hematoma to brain tissue (n=5). (**H**) The brain water content of the four groups at 3 days after ICH (n=6). Data in (D) and (E) are presented as the median (IQR) and data in (G) and (H) are shown as mean ± SEM. Kaplan-Meier survival analysis was performed to assess the data in (C). Kruskal-Wallis tests were performed for data comparisons in (D) and (E). One-way ANOVA was performed for data comparisons in (G) and (H). Statistical significance was denoted by *P < 0.05, **P < 0.01.

**Figure 3.**
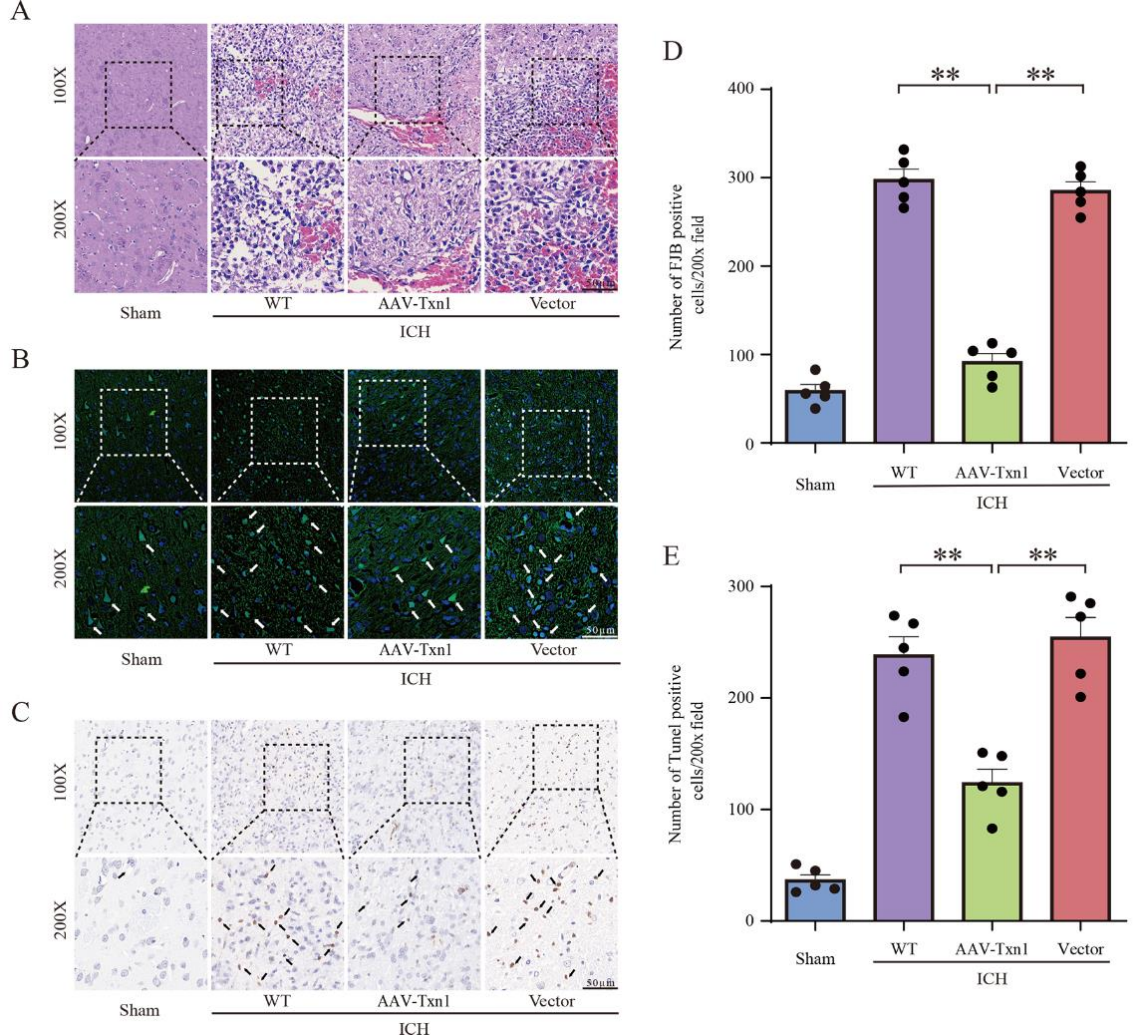
**Injection of AAV-Txn1 reduced the number of FJB^+^ cells and cell apoptosis after ICH**. (**A**) H&E staining in the sham rats, AAV-Txn1 injected rats, empty vector injected rats and WT rats at 3 days after ICH. Scale bar = 50μm (n=5). (**B**) Images showed FJB^+^ cells in the four groups. Scale bar = 50μm. (**C**) TUNEL staining showed cell apoptosis in the four groups. Scale bar = 50μm. (**D**) The number of FJB^+^ cells in the four groups (n=5). (**E**) Quantification of apoptotic cells in perihematomal brain tissues derived from the four groups (n=5). All pictures are selected from the perihematomal tissues at the striatal section. Data in charts were shown as mean ± SEM and analyzed by one-way ANOVA. Statistical significance was denoted by **P < 0.01.

### Overexpression of Txn1 attenuated brain edema and improved neurobehavioral recovery after ICH

Next, we studied the role of Txn1 in secondary injury following ICH. Three weeks before ICH surgery, we injected AAV-Txn1 or empty vector into rat brain (Fig. 2A) and used WB to confirm overexpression of Txn1 in the brain (Fig. 2B). First, we evaluated neurologic function and mortality rate after ICH. The results showed that AAV-Txn1 injection reduced the mortality rate of ICH rats (Fig. 2C). Furthermore, the rats treated with AAV-Txn1 exhibited significantly less neurologic deficit than control groups on days 3, 7, 14, and 21(Fig. 2D). For corner tests, all groups showed no difference after ICH on day 1. Overexpression of Txn1 corrected the turns on days 7, 14, and 21(Fig. 2E). Additionally, the hematoma volume (Fig. 2F, G) and brain water content (Fig. 2H) were reduced in the AAV-Txn1 group on day 3 after ICH. In all, over-expression of Txn1 promotes neurological recovery after ICH.

### Txn1 Suppressed Apoptosis and Inflammation Induced by ICH

**Figure 4.**
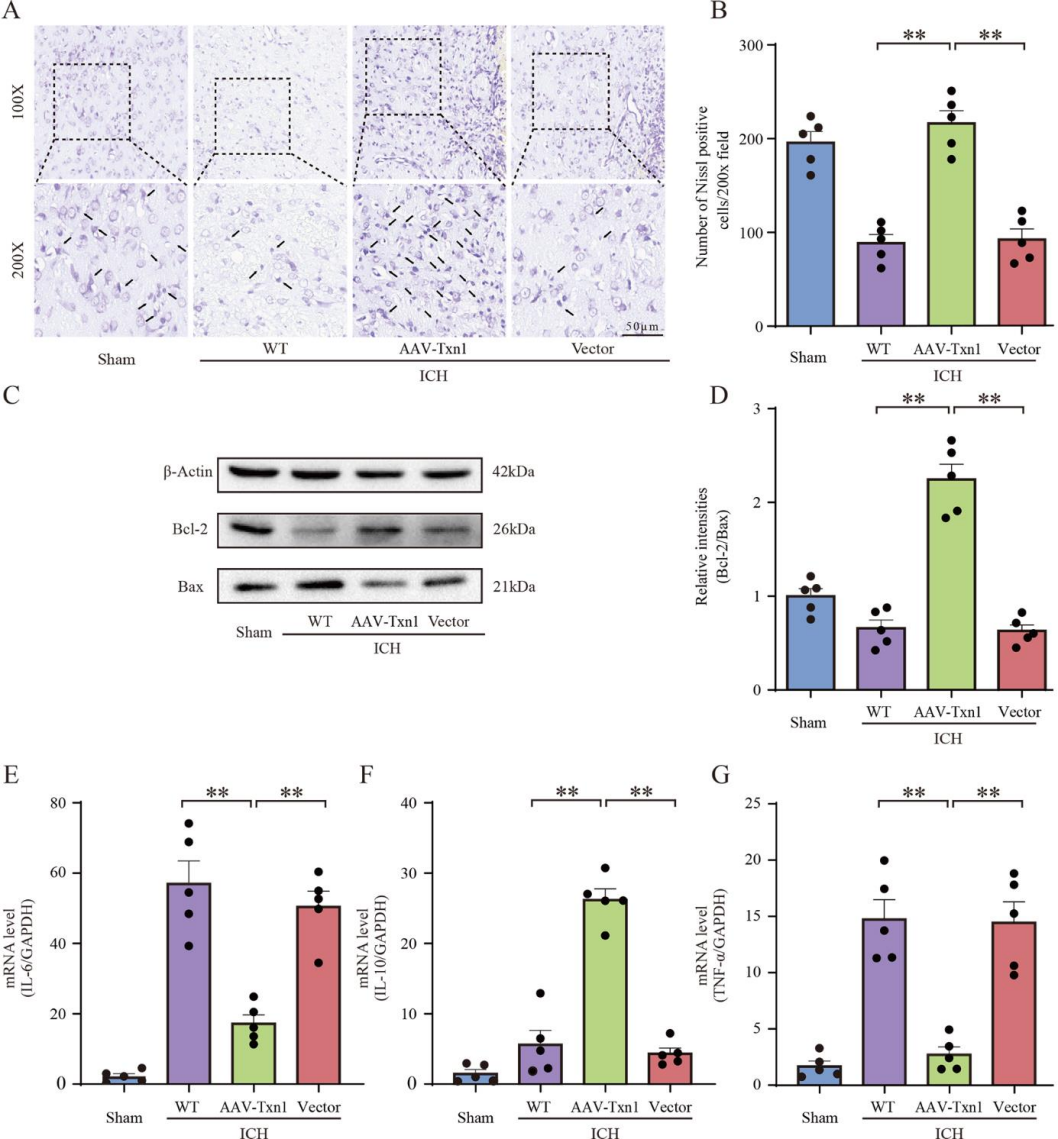
**Injection of AAV-Txn1 reduced cell apoptosis and inflammation of rats after ICH**. (**A**) Nissl staining of perihematomal brain tissues in sham rats, AAV-Txn1 injected rats, empty vector injected rats and WT rats at 3 days after ICH. Scale bar = 50μm. (**B**) The number of Nissl-positive cells was assessed (n=5). (**C**) Western blot showed the expression of Bcl-2 and Bax in the four groups (n=5). (**D**) The relative intensities of protein expression levels (Bcl2/Bax) in the four groups (n=5). (**E**) qRT-PCR showed the expression of *IL-6* in the four groups (n=5). (**F**) qRT-PCR showed the expression of *IL-10* in the four groups (n=5). (**G**) qRT-PCR showed the expression of *TNF-α* in the four groups (n=5). The pictures in (A) are selected from the perihematomal tissues at the striatum. Data in charts were shown as mean ± SEM and analyzed by one-way ANOVA. Statistical significance was denoted by **P < 0.01.

First, we performed H&E staining on striatal peri-hematomal tissue and found that injection of AAV-Txn1 reduced the number of infiltrated immune cells, and alleviated vacuolation and parenchymal loss (Fig. 3A). Next, we conducted FJB (Fig. 3B, D) and TUNEL staining (Fig. 3C, E) to detect damaged cells in striatal perihematomal tissue and found that the number of TUNEL^+^ and FJB^+^ cells was significantly reduced in the AAV-Txn1 group. We also found more Nissl-positive cells in the AAV-Txn1 rats (Fig. 4A, B). Further, we explored the expression of apoptosis-related proteins using WB and found that the level of Bcl-2/Bax increased in ICH rats injected with AAV-Txn1 (Fig. 4C, D). We also measured the levels of inflammatory factors and found a significant decrease in the mRNA levels of IL-6 and TNF-α and an increase of IL-10 in the AAV-Txn1 rats (Fig. 4E-G). Conclusively, the above results indicated that Txn1 could attenuate brain injury by inhibiting neuronal apoptosis and reducing inflammatory responses after ICH.

**Figure 5.**
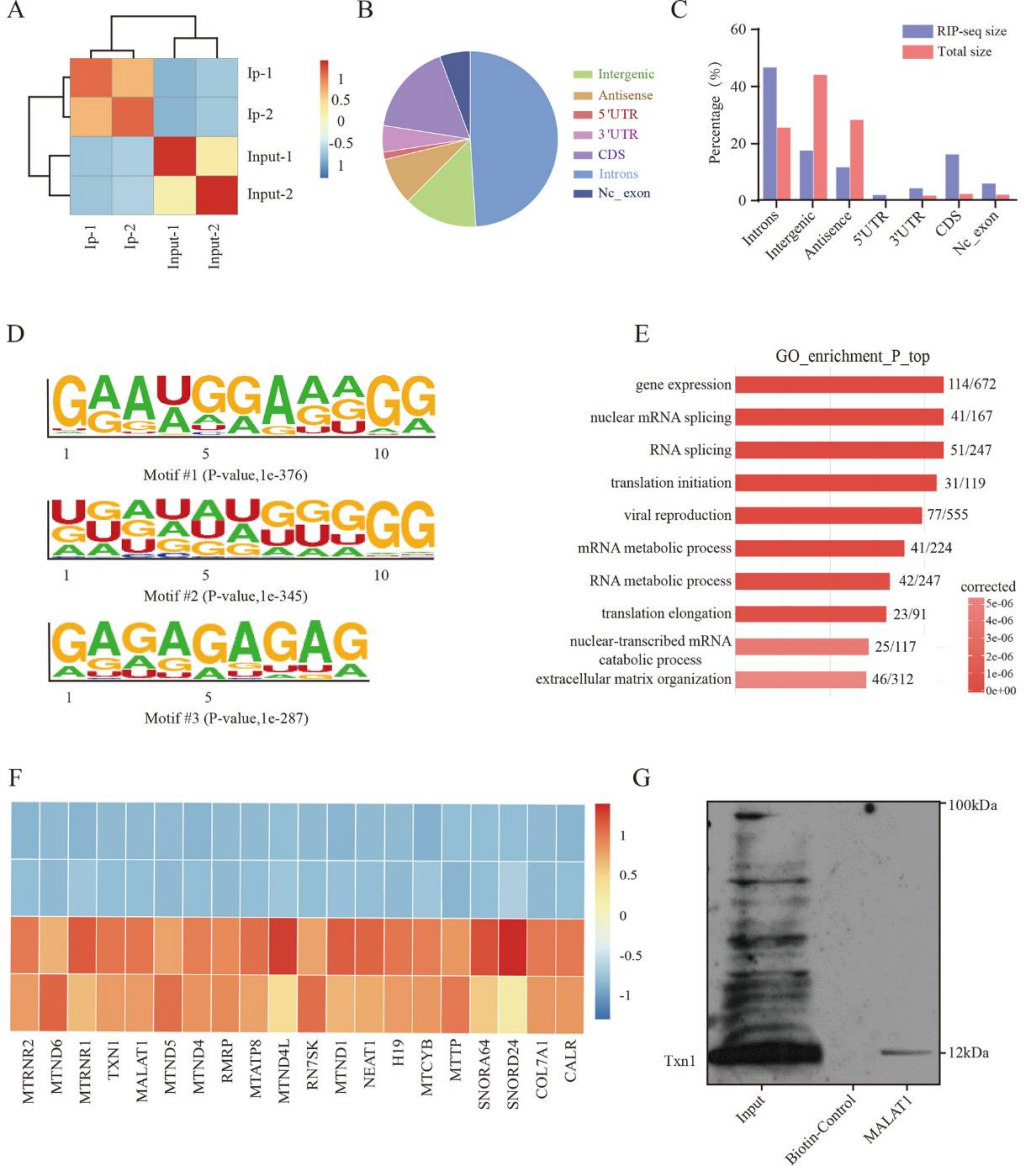
**The genome-wide landscape of Txn1 binding sites on RNA**. (**A**) The correlation of biological replicates between samples. (**B**) Txn1 RNA immunoprecipitation combined with RIP-seq showed peak distribution proportion of the Txn1 RIP-seq peaks. (**C**) The Txn1 RIP-seq peaks were predominantly enriched in the CDS region, 3´-UTR, 5´-UTR, and introns. All RIP-seq peaks were classified based on their distribution on the RNA elements and compared to the genomic background. (**D**) De novo motif analysis identified GA repeat and GA-enriched sequences as Txn1 binding motifs. (**E**) GO analysis of Txn1-dependent DEGs. The x-axis indicated the enrichment P-value on a -log10 scale, and the y-axis indicated terms. (**F**) Heat map showing the high abundance of RNA bound to Txn1 protein. The y-axis indicates the enrichment P-value on a -log10 scale, and the x-axis indicates terms. (**G**) Western blot was used to detect proteins combined with MALAT1 probe.

### Characterization of Txn1 Binding to Target RNA In vitro

We further adopted RIP-seq to identify all RNAs that could interact with Txn1 to investigate the mechanism by which Txn1 inhibited inflammation and apoptosis. First, we verified the consistency of the two Txn1 RIP-seq data replicates and the results showed good consistency (Fig. 5A). Next, we analyzed the regions associated with Txn1-bound RNAs and found that enrichment was mainly in the introns and CDs region (Fig. 5B, C). Such enrichment in introns indicates that the protein may bind to pre-mRNAs and affect RNAs’ stability [27]. Also, the enrichment in the 3' untranslated region (3'-UTR) and in 5'-UTR suggested that Txn1 was related to regulating RNA stability [28] and translation [29]. Furthermore, the de novo motif analysis results (Fig. 5D) indicated that Txn1 mainly bound to the AG-enriched region and may play a role in post-transcriptional gene regulation in multicellular organisms by affecting both the stability and translation of mRNAs via combining with target RNA, as the RNA-induced silencing complex (RISC) recognizes target mRNAs and most commonly results in translational inhibition or destabilization of the target mRNA [30, 31]. Finally, gene ontology (GO) analysis of the Txn1-bound RNAs suggested significantly enriched terms for gene expression (Fig. 5E). These results suggested that Txn1 may bind to related RNA to affect RNA stability and gene expression for modulating neuroprotective effects. To find out which RNA binds to Txn1 for suppressing apoptosis and inflammatory responses, we screened highly abundant Txn1-bound RNAs and found MALAT1 is one of them (Fig. 5F). The RNA pull-down analysis also proved the combination of Txn1 and MALAT1 (Fig. 5G). Previous studies showed that the lncRNA MALAT1 is involved in various pathological processes, which include inflammatory pathways, immune responses, and apoptosis, associated with secondary injury of ICH. Therefore, we speculate that the protective effect of Txn1 may be attributed to its binding to MALAT1 after ICH.

**Figure 6.**
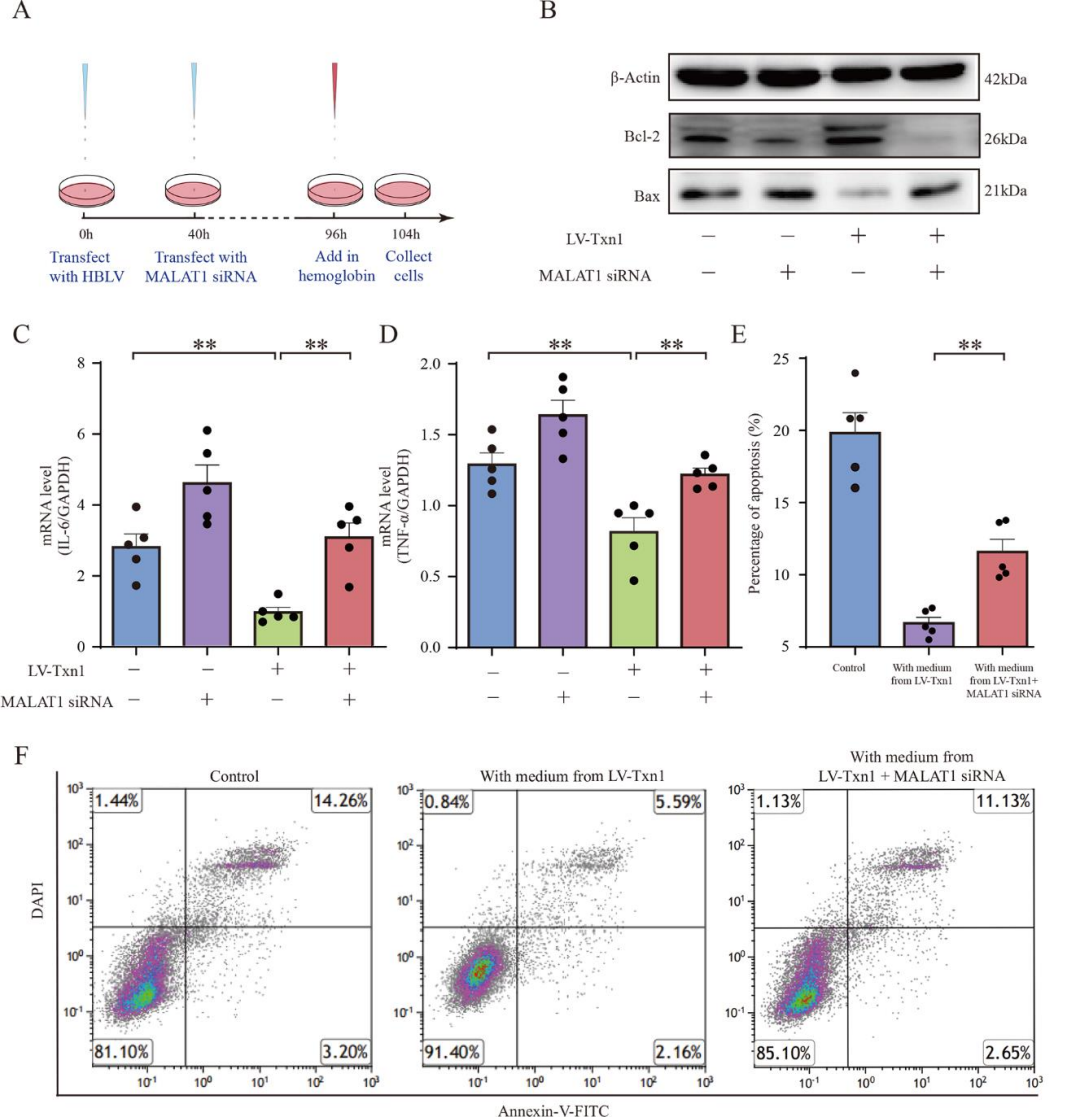
**Txn1 combined with MALAT1 and regulated inflammation and cell apoptosis**. (**A**) The workflow of in vitro experiments. (**B**) Western blotting analysis showed Bcl-2 and Bax levels in the empty vector transfected BV2 cells, MALAT1 siRNA transfected BV2 cells, LV-Txn1-transfected BV2 cells and LV-Txn1 + MALAT1 siRNA transfected BV2 cells. (**C**) The IL-6 levels of the four groups in the BV2 cells (n=5). (**D**) The TNF-α levels of the four groups in the BV2 cells (n=5). (**E**) The percentage of apoptotic neurons cultured with media from LV-Txn1-transfectecd BV2 cells and LV-Txn1+MALAT1 siRNA transfected BV2 cells (n=5). (**F**) Flow cytometric results of apoptotic neurons cultured with media from LV-Txn1-transfectecd BV2 cells and LV-Txn1+MALAT1 siRNA transfected BV2 cells. Data in charts were shown as mean ± SEM and analyzed by one-way ANOVA. Statistical significance was denoted by **P < 0.01.

### Txn1 Attenuated Inflammation and Apoptosis by Affecting MALAT1 Expression

Next, we examined the changes of MALAT1 after ICH and found that MALAT1 was significantly reduced compared with the sham group (Supplementary Fig. 2B). We speculated that Txn1 binds to MALAT1 and enhances its stability. Txn1 expression was reduced after ICH, and as a result, MALAT1 stability was also reduced, leading to attenuated inhibition of inflammation and apoptosis. We conducted the following experiments to test this hypothesis (Fig. 6A): First, we transfected BV2 cells with LV carrying Txn1 (LV-Txn1) to overexpress Txn1 in BV2 cell line (Supplementary Fig. 2C, D). Further, we used qRT-PCR to detect MALAT1 levels, and the results verified our speculation. MALAT1 levels were significantly increased in the BV2 cells transfected with LV-Txn1 (Supplementary Fig. 2E). We further transfected MALAT1 siRNA into LV-Txn1 treated BV2 cells, and the qRT-PCR results confirmed that the RNA level of MALAT1 was significantly reduced (Supplementary Fig. 2F). Next, we added hemoglobin to the culture medium to simulate ICH microenvironment in vitro and detected inflammatory factors and apoptosis-related proteins in each group. Our results showed that IL-6 and TNF-α levels were significantly increased in the LV-Txn1+MALAT1 siRNA transfected group compared with the LV-Txn1 transfected group (Fig. 6C, D). In contrast, Bcl-2/Bax levels were significantly decreased (Fig. 6B). These results suggested that inhibiting MALAT1 expression could reverse Txn1-induced neuroprotective effect in ICH rat. Finally, we incubated neurons with media from the LV-Txn1-transfected and LV-Txn1+MALAT1 siRNA tranfected groups. The flow cytometry results showed that transfection of Txn1 significantly improved neuronal survival, while knock down of MALAT1 reversed this protective effect (Fig 6E, F). The above results showed that Txn1 plays an anti-inflammatory and anti-apoptotic role by binding to MALAT1.

## DISCUSSION

RNA-binding proteins play critical roles in post-transcriptional regulation and various pathophysiological processes [32, 33]. However, their role in ICH has rarely been investigated. Here, we found that Txn1 binds to MALAT1 to reduce inflammation and apoptosis, and further alleviates neurological deficits after ICH. These results suggest that Txn1 can be employed as a therapeutic target to inhibit secondary injury after ICH. Here, we found that the Txn1 mRNA levels significantly increased after ICH, although the protein levels significantly decreased (Supplementary Fig 2A). We suspect that this difference may be attributed to the following factors: 1) Txn1 consumption increased after ICH. Although the transcription level increased, the total protein level remained lower than normal. 2) Txn1 ubiquitination degradation: previous studies showed that Txn1 can be degraded through a methylglyoxal (MGO)-mediated 5’AMP-activated protein kinase (AMPK)-dependent pathway [25, 26]. We speculated that the decrease in Txn1 might also be related to enhanced ubiquitination after ICH. 3) Post-transcriptional regulation: although the Txn1 mRNA levels significantly increased, it may be affected by post-transcription regulation and cannot be translated into a protein. Therefore, the trend between unbalanced Txn1 RNA and protein requires further experimental studies and verification. Moreover, immunofluorescence staining results first revealed that Txn1 was expressed in the neurons and microglia. This cellular localization proposes that Txn1 may play a vital role in neuronal protection and secondary injury induced by ICH. Furthermore, we found that overexpression of Txn1 reduced rat mortality, promoted neurological recovery, and reduced brain edema and hematoma volume after ICH. These in vivo experiments demonstrated that Txn1 plays an important role in promoting neurological recovery after ICH.

Previous studies revealed that Txn1 inhibits the apoptosis and inflammatory process through inhibiting stress kinase-1 (ASK1)-dependent pathway [34], arresting thioredoxin-interacting protein/nod-like receptor protein 3 (TXNIP/NLRP3) inflammasome activation [35], and down-regulating the nuclear factor-kappa B (NF-κB) pathway [36]. The western blots and qRT-PCR results in this study also proved it. Moreover, our in vitro experiments revealed that Txn1 acts as an RBP, which provides a new function for anti-apoptosis and inflammation.

Although numerous studies have shown that Txn1 is a RBP [37, 38], which RNA it binds to and the changes that occur in these RNAs remain unknown. Therefore, we investigated the properties of Txn1 from a genome-wide perspective and identified MALAT1, a highly abundant Txn1-bound RNA, for further research. The RIP-seq together with in vitro experiments suggested that Txn1 may bind to MALAT1 and enhance its stability, thus attenuating inflammation and apoptosis, and improving neuronal survival. In summary, we first revealed that Txn1 exerts neuroprotective effects after ICH from the perspective of RNA binding proteins. This provides a new perspective for the research on the role of Txn1.

## Supplementary Materials

The Supplementary data can be found online at: www.aginganddisease.org/EN/10.14336/AD.2023.0507.


