# Supplementary material for: Thioredoxin1 Binding Metastasis-Associated Lung Adenocarcinoma Transcript 1 Attenuates Inflammation and Apoptosis after Intracerebral Hemorrhage
Source: Aging Dis. 2024 May 7;15(3):1384–97. doi: 10.14336/AD.2023.0507 (PMC11081159; doi:10.14336/AD.2023.0507)
Supplement: Supplementary file 1 [file AD-15-3-1384-s.pdf]

SUPPLEMENTARY DATA

Thioredoxin1 Binding Metastasis-Associated Lung Adenocarcinoma Transcript 1 Attenuates Inflammation and Apoptosis after Intracerebral Hemorrhage

Ru Chen, Qi Xie, Lexing Xie, Jiacheng Huang, Linlin Hu, Hui Lu, Peixia Shi, Qian He, Qin Zhang, Changxiong Gong, Shuang Zhang, Bingqiao Wang, Guoqiang Yang, Qingwu Yang

© 2023. Chen R et al. Published online at http://www.aginganddisease.org/EN/10.14336/AD.2023.0507

SUPPLEMENTARY DATA

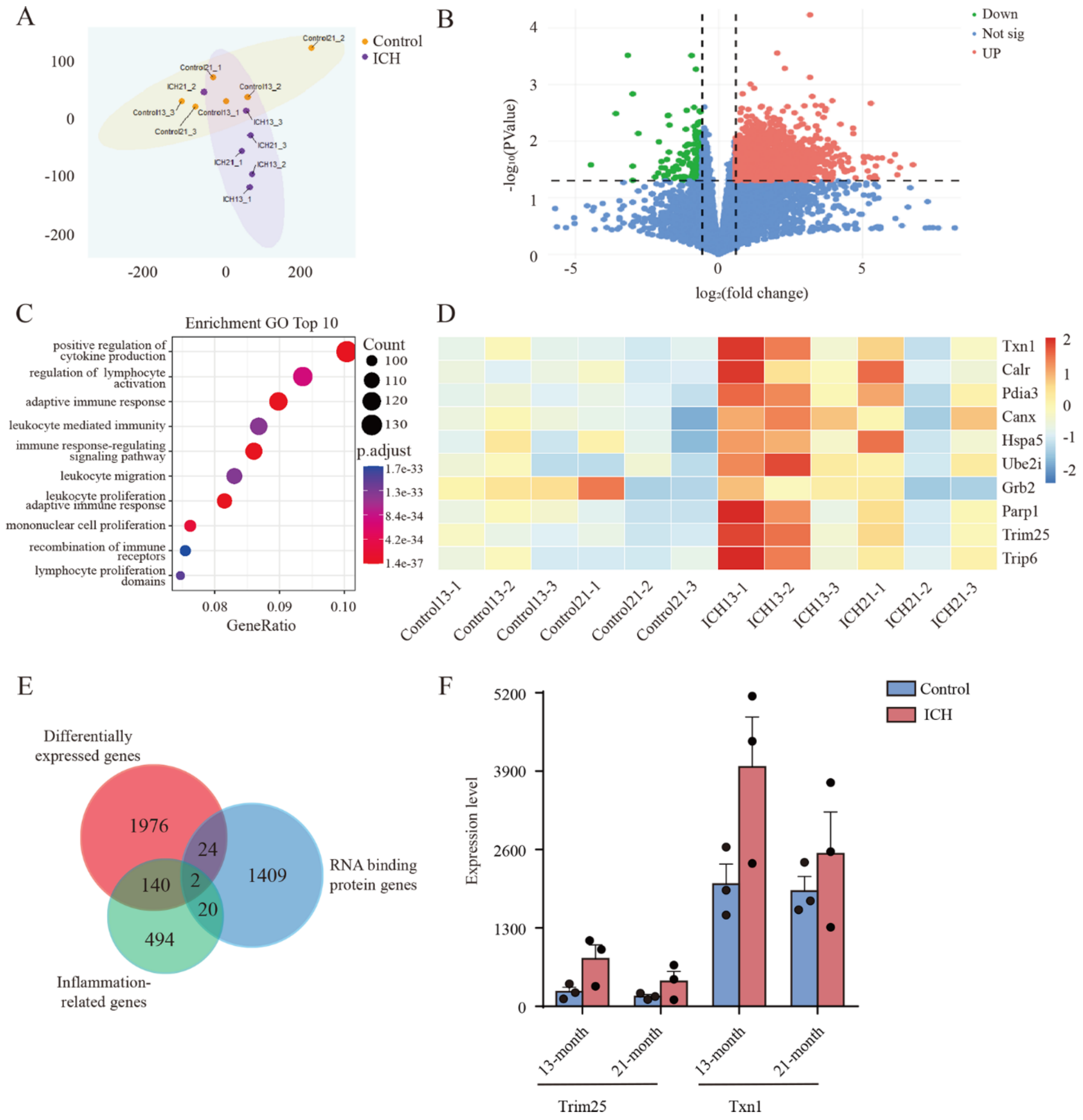

Supplementary Figure 1. **Txn1 was the most differentially expressed RBP after ICH. (A)** PCA of the two groups of rats: 13 represents 13-month-old rats and 21 represents 21-month-old rats. **(B)** Volcano Plot of differentially expressed genes after ICH. **(C)** GO analysis of up-regulated differentially expressed genes. The x-axis indicated the enrichment *P*-value on a -log10 scale, and the y-axis indicated terms. **(D)** The heatmap of the top ten expressed RBPs between ICH and control. The x-axis indicated the enrichment *P*-value on a -log10 scale, and the y-axis indicated terms. 13 represents 13-month-old rats and 21 represents 21-month-old rats. **(E)** The Venn diagram of differentially expressed genes, RNA binding protein genes, and inflammation-related genes analyzed by RNA-seq. **(F)** The expression level of Trim25 and Txn1 in rats after ICH (n = 3), 13-month indicates 13-month-old rats and 21-month indicates 21-month-old rats.

© 2023. Chen R et al. Published online at http://www.aginganddisease.org/EN/10.14336/AD.2023.0507

SUPPLEMENTARY DATA

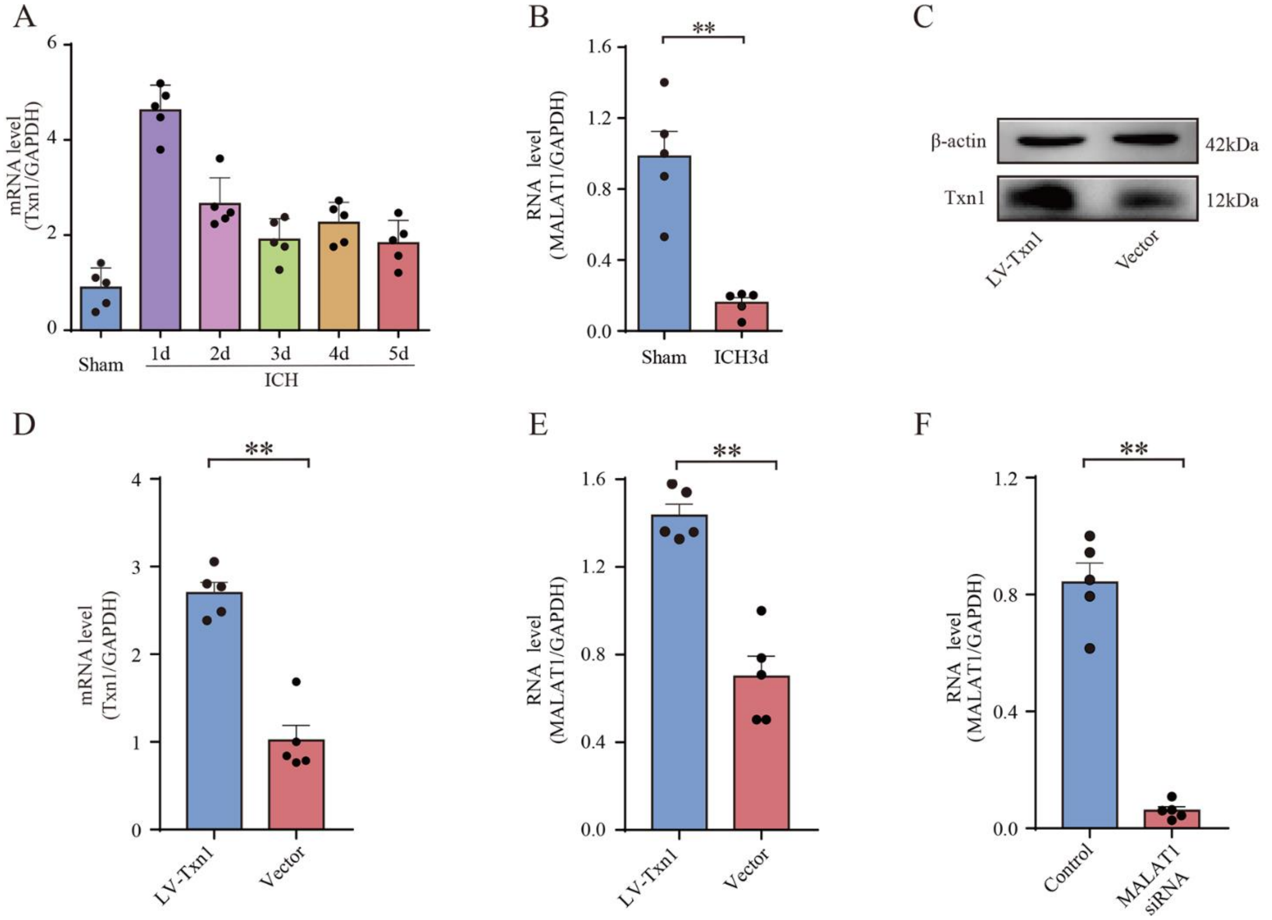

Supplementary Figure 2. **Expression of Txn1 and MALAT1. (A)** The mRNA level of Txn1 confirmed by qRT-PCR. **(B)** The RNA level of MALAT1 in rats confirmed by qRT-PCR after ICH (n=5). **(C)** The Western blotting analysis of Txn1 in BV2 cells. **(D)** qRT-PCR showed Txn1 expression in BV2 cells (n=5). **(E)** qRT-PCR showed the RNA level of MALAT1 (n=5). **(F)** qRT-PCR showed the RNA level of MALAT1 in control group and MALAT1 siRNA transfected BV2 cells (n=5). Mann-Whitney U test was performed for comparisons between the two groups in (F) and Unpaired t-tests were performed for other comparisons between the two groups. Statistical significance was denoted by **P < 0.01.

© 2023. Chen R et al. Published online at http://www.aginganddisease.org/EN/10.14336/AD.2023.0507

SUPPLEMENTARY DATA

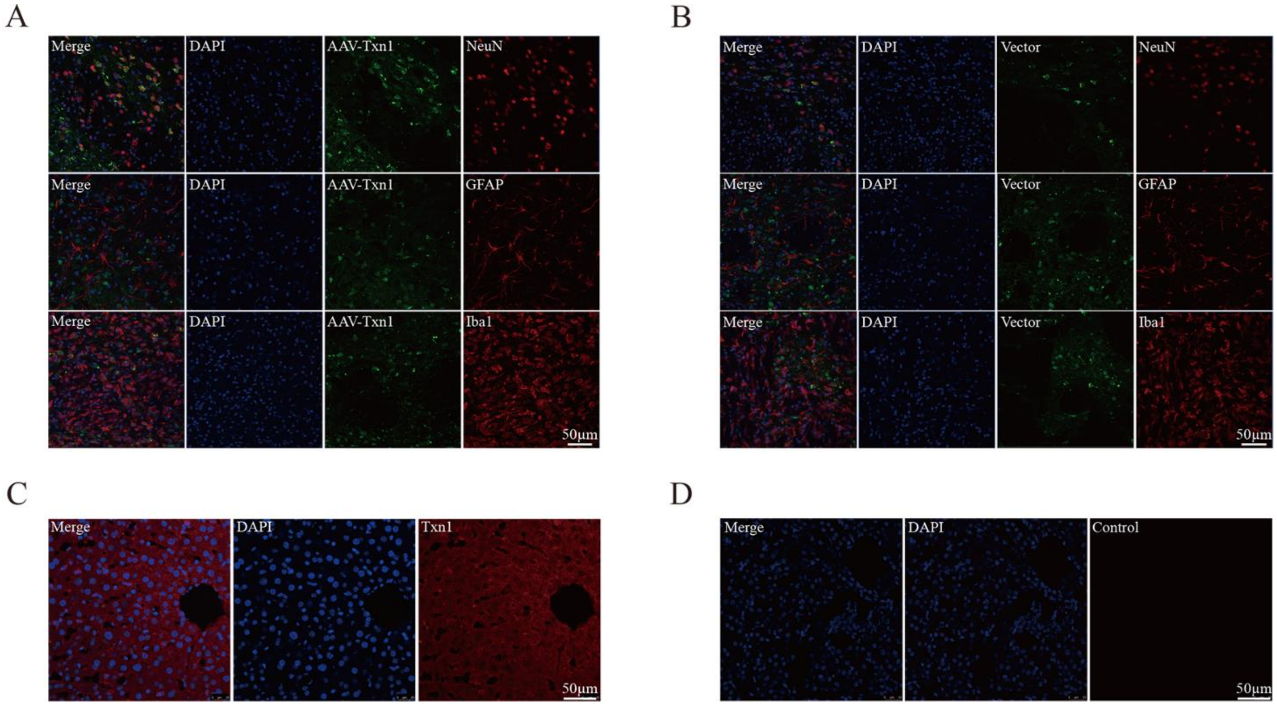

Supplementary Figure 3. Expression of Txn1 in rats. (A) Immunostaining results showed co-localization of AAV-Txn1 with neurons (NeuN), astrocytes (GFAP) and microglia (Iba1) in AAV-Txn1 injected rats at 3 days after ICH. Scale bar = 50 µm. **(B)** Immunostaining results showed co-localization of empty vector with neurons (NeuN), astrocytes (GFAP) and microglia (Iba1) in empty vector injected rats at 3 days after ICH. Scale bar = 50 µm. **(C)** Immunostaining results showed Txn1 expression in the liver tissue. Scale bar = 50 µm. **(D)** Immunostaining results showed Txn1 expression in the negative control. Scale bar = 50 µm.

© 2023. Chen R et al. Published online at http://www.aginganddisease.org/EN/10.14336/AD.2023.0507
